# A Rare and Unique Complication of Uncontrolled Type 2 Diabetes Mellitus: A Case Report and Literature Review of Spontaneous Diabetic Myonecrosis

**DOI:** 10.7759/cureus.37099

**Published:** 2023-04-04

**Authors:** Adejoke M Johnson, Zin Thawdar Oo, Thar Sann Oo, Damion A Hunter, Zin M Htet, Vishal Reddy Bejugam, Gabriel Purice

**Affiliations:** 1 Medicine, Jacobi Medical Center/North Central Bronx Hospital, Bronx, USA; 2 Internal Medicine, Jacobi Medical Center/North Central Bronx Hospital, Bronx, USA; 3 Internal Medicine, Albert Einstein College of Medicine, Jacobi Medical Center, Bronx, USA; 4 Internal Medicine, Jacobi Medical Center, Bronx, USA; 5 Internal Medicine, James J. Peters Veterans Affairs (VA) Medical Center, Bronx, USA; 6 Internal Medicine, North Central Bronx Hospital, Bronx, USA

**Keywords:** uncontrolled diabetes mellitus, myositis, myonecrosis, hemoglobin a1c, muscle infarction

## Abstract

There are many microvascular and macrovascular complications regarding uncontrolled diabetes mellitus (DM). Among them, diabetes myonecrosis is one of the complications but rarely seen in the uncontrolled DM patient population. Here, we present a rare case of DM myonecrosis in a patient with elevated hemoglobin A1c (HbA1c) of 18.2% and discuss the literature review of diabetes myonecrosis. A 48-year-old male with hypertension and uncontrolled type 2 diabetes mellitus (T2DM) with hemoglobin A1c of 18.2% presented with progressive swelling and pain in the right thigh for two days. Physical examination demonstrated swollen and tense tender right thigh with a circumference five inches larger than the left. Computed tomography (CT) and magnetic resonance imaging (MRI) results revealed severe myositis of the right leg, likely myonecrosis, and associated fascial edema/fasciitis. The patient was also complicated with diffuse anasarca, which was corrected with albumin transfusion and furosemide. Aspirin and lisinopril were also started for antithrombotic and cardioprotective effects. The right thigh swelling improved, and the patient could ambulate with supportive measures and regular physical therapy (PT). He was discharged home after 45 days of hospitalization. Diabetic myonecrosis is a rare condition and hence is underdiagnosed. In patients with uncontrolled diabetes, especially with diabetic complications, physicians should have high clinical suspicion to diagnose diabetic myonecrosis when patients present with an acute unilateral painful swollen limb. Our case highlights the complicated course of diabetes myonecrosis with anasarca, improved with supportive measures.

## Introduction

Diabetes is associated with vascular complications, typically microvascular complications such as retinopathy, nephropathy, and peripheral neuropathy and macrovascular complications such as cardiovascular disease, cerebrovascular disease, and peripheral vascular disease. Poor glycemic control increases the risk of some of these complications, especially microvascular complications, and diabetic myonecrosis is one of them but relatively rare [1]. Diabetic myonecrosis is a spontaneous skeletal muscle infarction frequently reported in patients with poorly controlled type 1 and 2 diabetes mellitus (DM) with a sub-optimized hemoglobin A1c (HbA1c) level [1].

## Case presentation

**Figure 1:**
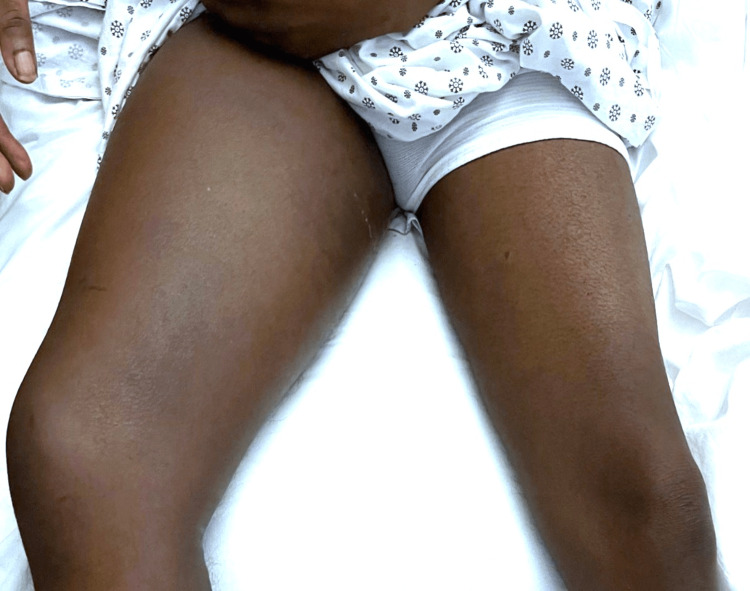
Right thigh swelling

A 48-year-old male with a history of hypertension and poorly controlled type 2 diabetes mellitus (T2DM) presents to the emergency department with complaints of progressive right thigh swelling and pain (Figure 1).

The patient had been experiencing intermittent swelling and stabbing right thigh pain for two months, which had worsened over the course of two days and resulted in difficulty with ambulation. He also reported intermittent chills and shortness of breath due to intractable pain, numbness, and tingling of both upper and lower extremities. He denied a history of falls, trauma, injury to his lower extremities, fever, night sweat, nausea, vomiting, abdominal pain, change of urinary or bowel habits, and weakness of his arms and legs. The patient was diagnosed with T2DM 15 years ago and missed insulin treatment for a year before he came to the hospital. Physical examination revealed swollen and tense right thigh with tenderness, and the thigh circumference was five inches larger than the left. Vitals were significant for blood pressure of 170/74 mmHg. Laboratory results were significant for elevated glucose, hemoglobin A1c, leukocytosis, thrombocytosis, and increased creatinine kinase (Table 1).

**Table 1:** Laboratory investigations BNP, brain natriuretic peptide; CPK, creatine phosphokinase

Test	Result	Normal range
Hemoglobin A1c (HbA1c)	18.2%	4%-5.6%
Glucose	702 mg/dL	70-99 mg/dL
Anion gap	6.3 mEq/L	<14 mEq/L
Sodium	133 mEq/L	135-145 mEq/L
Corrected sodium	143 mEq/L	135-145 mEq/L
Potassium	5.1 mEq/L	3.5-5 mEq/L
Blood urea nitrogen (BUN)	26 mg/dL	5-26 mg/dL
Creatinine	1 mg/dL	0.5-0.9 mg/dL
Lipase	519 U/L	7-60 U/L
Troponin	0.081 ug/L	0.000-0.090 ug/L
Pro-BNP	332.9 pg/mL	1-125 pg/mL
White blood cell count	12.10/NL	3.9-10.6/NL
Hemoglobin	9.4 g/dL	13.5-17.5 g/dL
Platelet count	579/NL	150-440/NL
CPK	477 U/L	5-150 U/L

**Figure 2:**
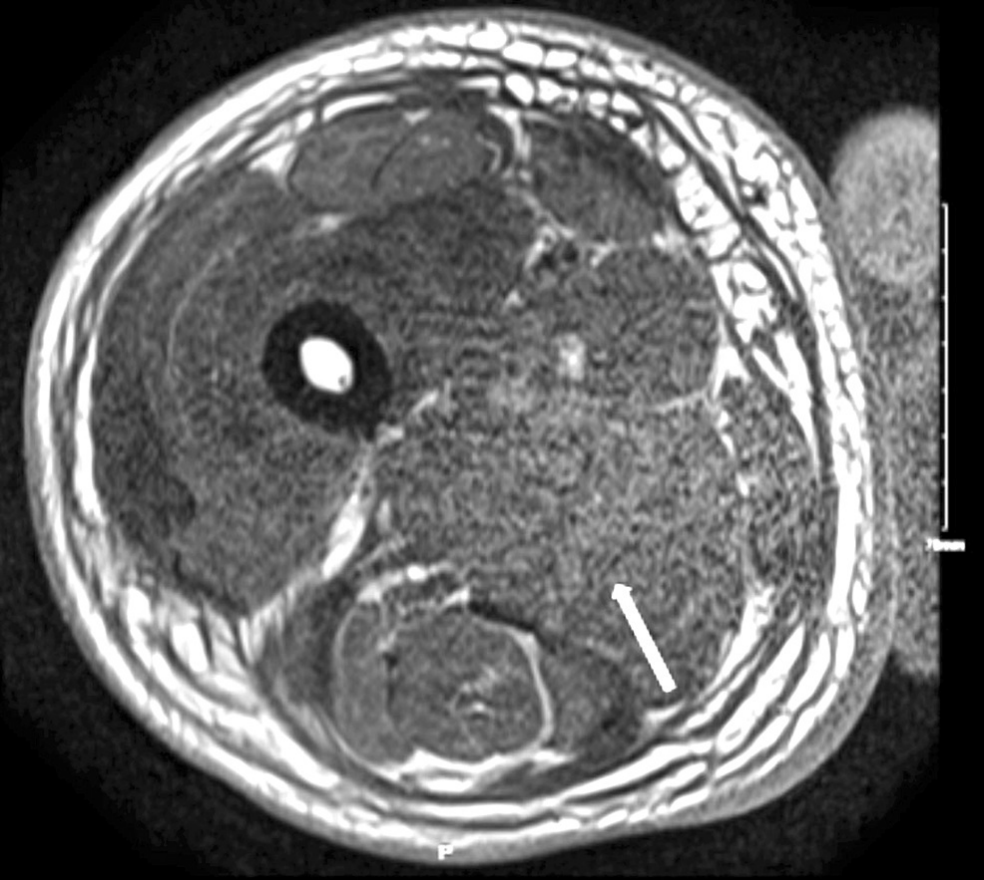
MRI of the right thigh in the axial section without contrast demonstrating the loss of internal architecture of muscle, showing severe myositis MRI: magnetic resonance imaging

**Figure 3:**
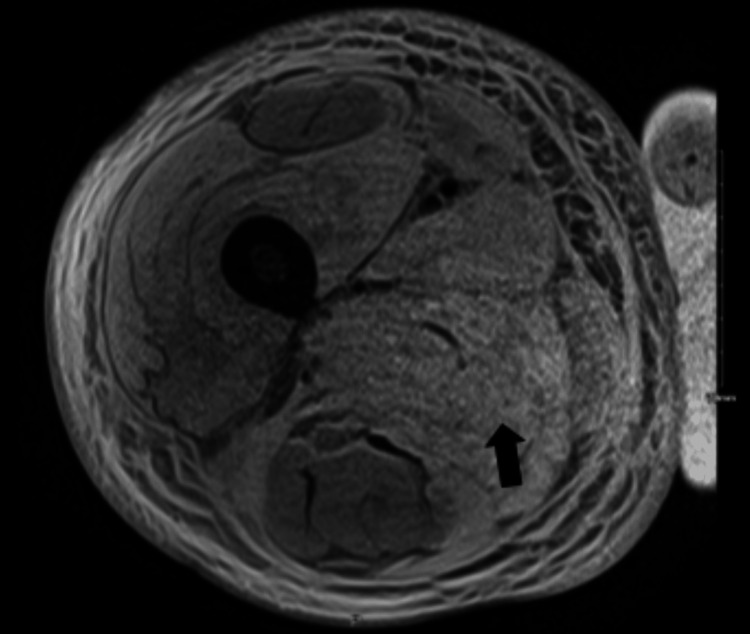
MRI of the right thigh in axial section with contrast demonstrating the loss of internal architecture of muscle with contrast enhancement showing severe myositis MRI: magnetic resonance imaging

**Figure 4:**
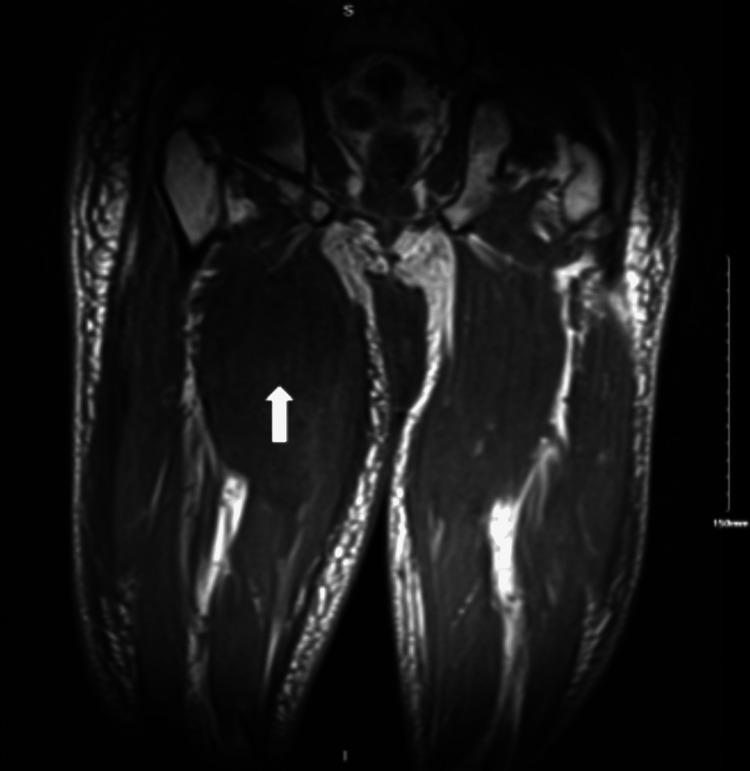
MRI of the right thigh in the coronal section without contrast showing severe myositis with associated superficial and deep fasciitis and the areas of myonecrosis MRI: magnetic resonance imaging

**Figure 5:**
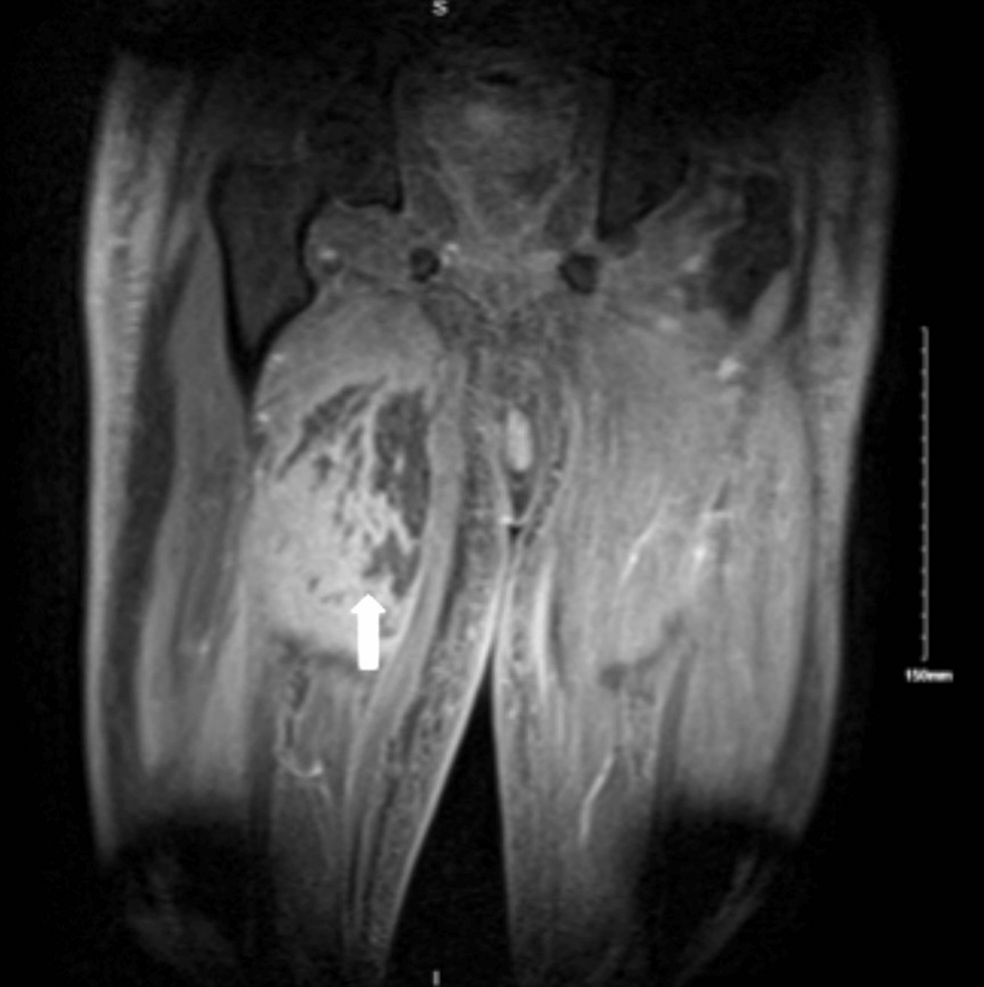
MRI of the right thigh in coronal view with contrast showing severe myositis with associated superficial and deep fasciitis and the areas of myonecrosis MRI: magnetic resonance imaging

Blood cultures obtained throughout hospitalization had no growth; the antinuclear antibody (ANA)-extractable nuclear antigen (ENA) antibody panel was positive only for the SCL-70 antibody. All rheumatological workups were negative. Bilateral lower extremity ultrasound showed subcutaneous edema without acute deep vein thrombosis. A computed tomography (CT) of the abdomen and pelvis also demonstrated soft tissue swelling and fat stranding, most prominent within the right thigh. CT angiogram of the right thigh was done to rule peripheral vascular disease, which revealed diffuse subcutaneous fat stranding and edema in the interfascial plane of the right thigh, relative hypo-enhancement of the adductor magnus muscle suggestive of myositis with likely myonecrosis, and associated fascial edema/fasciitis. Magnetic resonance imaging (MRI) findings of the right femur were also compatible with severe myositis with associated superficial and deep fasciitis and the area of myonecrosis of the right leg (Figures 2-5). At the same time, left thigh MRI results revealed significantly less pronounced myositis than the right thigh.

General surgery was consulted for high suspicions of compartment syndrome but suggested that no surgical intervention was needed. The patient was started on aspirin and pain control, and physical therapy (PT) was started intermittently when his thigh swelling and pain had improved. Endocrinology was consulted for glycemic control. The patient was symptomatically treated with pain management, physical therapy as tolerated, and glycemic control with three units of basal and corrective insulin scales. Unfortunately, on day 8, the hospital course was complicated by diffuse anasarca in the setting of low albumin levels and intravenous (IV) fluid. The nephrology team was consulted, and they recommended starting with IV Lasix 40 mg twice daily, hydrochlorothiazide 12.5 mg daily, and 25 g of 25% albumin transfusion for two days. The patient became euvolemic on day 11 of hospitalization with gradual resolution of myositis.

Since the patient also had nephrotic range proteinuria of 4.6 g, a renal biopsy was done to rule out other causes of nephrotic range proteinuria. The renal biopsy results show moderate nodular diabetic glomerulosclerosis, mild tubular atrophy, and interstitial fibrosis, and he was eventually started on lisinopril 40 mg and Lasix 20 mg for fluid overload. He spent a total of 45 days in the hospital due to poor pain management, diffuse anasarca secondary to nephrotic syndrome that required further renal workup, and symptomatic COVID-19 infection. Some social factors also affected the patient's safe discharge during the last 10 days of hospitalization. After 45 days in the hospital, he was able to ambulate with assistance and was discharged with home physical therapy.

## Discussion

Diabetes mellitus is a complex metabolic disease associated with multiple systemic complications, primarily when the condition is poorly controlled. The complications can be acute or chronic, but chronic complications are associated with higher morbidity and mortality in diabetes mellitus [2]. Chronic complications can be divided into microvascular and macrovascular [3]. The report from 1999-2004 National Health and Nutrition Examination Survey (NHANES) revealed that microvascular complications, including peripheral neuropathy, retinopathy, and nephropathy, are more common compared to macrovascular complications such as coronary artery disease, heart failure, and stroke [4]. Other microvascular complications of diabetes mellitus not routinely discussed in the literature are diabetic myonecrosis and diabetic amyotrophy.

Diabetic myonecrosis is also known as diabetic skeletal muscle infarction, aseptic or ischemic myonecrosis, and tumoriform focal muscular degeneration. It was first reported in 1965 as a rare microangiopathic complication of poorly managed type 1 or type 2 diabetes [5]. A systematic review was done in 2003, including 115 patients with diabetic myonecrosis. It was observed that the disease is commonly diagnosed in middle-aged females (61.53%) and patients with an average A1C of 9% [5].

The exact pathophysiology of diabetic myonecrosis is not well established, although several theories suggest that vasculopathic changes in the muscles drive it. These changes are similar to the pathogenesis of coronary artery disease induced by prolonged exposure to hyperglycemia. It is highlighted as a significant factor in accelerated atherosclerosis in microvascular endothelium leading to ischemia and reperfusion injury [6]. The mechanisms for accelerated atherosclerosis occur through the nonenzymatic glycosylation of lipids and proteins in blood vessels, oxidative stress, and pro-inflammatory response. Hypercoagulation by alterations in the coagulation-fibrinolysis system has also been involved in the pathophysiology of diabetic myonecrosis [1,6].

The primary clinical presentation of diabetic myonecrosis is ipsilateral proximal lower limb acute or subacute pain with muscle swelling, weakness, and tenderness without a history of inciting factors. A systematic review analyzed 126 cases of diabetic myonecrosis in July 2014 from database inception, which revealed that thigh swelling was the most commonly reported affected site [7]. Diabetic myonecrosis usually has a mean duration of symptoms ranging from one day to 14 weeks [5]. The frequency of reoccurrence is still undetermined, although case reports reveal that the symptoms tend to recur in a different location. About 93% of patients that develop diabetic myonecrosis frequently have other concomitant diabetic complications, commonly diabetic nephropathy.

Complications of diabetic myonecrosis are uncommon but are usually spontaneous compartment syndrome and superimposed infection. The laboratory findings of diabetic myonecrosis stem from the disease's inflammatory process, which is indicated by elevated creatine kinase and erythrocyte sedimentation rate. Elevated white blood cells (WBCs) have also been commonly seen in multiple cases. An MRI scan is usually recommended for further evaluation [8]. It typically reveals signal intensity in the affected intramuscular and subcutaneous tissues, a hyperintense signal on T2-weighted imaging, and an isointense to hypointense signal on T1-weighted images associated with inflammatory changes and edema. The clinical manifestations and diagnostic findings of diabetic myonecrosis overlap with other etiologies; therefore, a diagnosis can only be established by excluding other etiologies such as pyomyositis, spontaneous gangrenous myositis, nontraumatic clostridial myonecrosis, and necrotizing fasciitis. A tissue biopsy is the definitive diagnostic test, but it is not often recommended for diagnosis, particularly in patients with concomitant diabetes complications, long-standing diabetes, and the absence of other probable etiologies [8]. Other tests, such as Doppler ultrasound, can rule out differential diagnosis of deep venous thrombosis or bacterial infection of the affected muscle.

The recorded incidence of diabetic myonecrosis has been limited since its discovery. Hence, standardized treatment management has yet to be established. It has been noted that there is a spontaneous resolution within 8-13 weeks in patients after bed rest and analgesic use [1,9]. Treatment for diabetic myonecrosis targets symptomatic management with rest, optimal glycemic control, nonsteroidal anti-inflammatory drug (NSAID) therapy, and low-dose aspirin.

NSAID can be used for short-term analgesia; however, risk has to be weighted with benefits since diabetes muscle infarction is associated with poorly controlled DM and is likely associated with conditions such as renal impairment and cardiovascular disease. Low-dose aspirin is initiated if there is no contraindication. Aspirin is used to prevent infarct recurrence, probably due to improvement in the dysfunctional state of the epithelium, although evidence is lacking. Other antiplatelet agents, such as clopidogrel, can be used for patients with aspirin allergies. The combination of NSAID and antiplatelet increases gastrointestinal bleeding risk, and one must be mindful of its usage. Physiotherapy usage is debatable; physical therapy (PT) is normally avoided in the acute stage of diabetic myonecrosis because it could prolong the recovery phase, but you also do not want the patient to be deconditioned; hence, PT Is mostly advised after discharge and has been proven beneficial in post-discharge care [10].

The long-term outlook of patients diagnosed with diabetic myonecrosis is poor as the disease indicates severe end-organ damage from the progression of the underlying diabetes mellitus [10]. Diabetes myonecrosis resolves over 5.5 weeks to 13 weeks, depending on the level of therapy initiated; with antiplatelet agents and anti-inflammatory drugs, the time frame for recovery is 5.5 weeks versus rest and analgesia at eight weeks and surgical excision at 13 weeks of recovery period. With resolution, the recurrence rate is approximately 40%. The long-term outcome tends to be poor due to five-year mortality from superimposed diabetic complications [11].

## Conclusions

Diabetic myonecrosis is a rare condition and hence is underdiagnosed. In patients with uncontrolled diabetes, especially with diabetic complications, physicians should have high clinical suspicion to diagnose diabetic myonecrosis when patients present with an acute unilateral painful swollen limb. However, other more common etiologies for acute unilateral painful swollen limbs such as deep vein thrombosis and cellulitis or different infectious etiologies will need to be ruled out using imaging techniques. Early noninvasive diagnosis is possible with MRI. Treatment involves symptomatic management and prevention of recurrence with low-dose aspirin and optimal glycemic control. The diagnosis, however, remains a marker of severe uncontrolled diabetes with a poor prognosis.
